# Longitudinal observation, evaluation and interpretation of coping with mental (emotional) health in low vision rehabilitation using the Dutch ICF Activity Inventory

**DOI:** 10.1186/s12955-014-0182-4

**Published:** 2014-12-24

**Authors:** Janna E Bruijning, Ger HMB van Rens, Mark Fick, Dirk L Knol, Ruth MA van Nispen

**Affiliations:** Department of Ophthalmology, VU University Medical Center, PO Box 7057, 1007 MB Amsterdam, The Netherlands; EMGO Institute for Health and Care Research (EMGO+), VU University Medical Center, Van der Boechorststraat 7, 1081 BT Amsterdam, The Netherlands; HU University of Applied Sciences Utrecht, PO BOX 85182, 3508 AD Utrecht, The Netherlands; Department of Ophthalmology, Elkerliek Hospital, Wesselmanlaan 25, 5707 HA Helmond, The Netherlands; Department of Epidemiology and Biostatistics, VU University Medical Center, PO Box 7057, 1007 MB Amsterdam, The Netherlands

**Keywords:** Low vision, Rehabilitation, D-AI, ICF, Activities and participation, Patient-centered, Mental health, Acceptance, Fatigue, Handle feelings

## Abstract

**Background:**

Since there is evidence that mental health aspects (such as depression) may inhibit an optimal rehabilitation outcome, there is growing interest in the psychosocial aspects of vision loss as part of rehabilitation. The purpose of this study is to provide more insight into the construct validity and (longitudinal) interpretation of goals related to ‘Coping with mental (emotional) health aspects’ which are part of the recently developed ‘Dutch ICF Activity Inventory (D-AI). Moreover, the data allowed to provide some insight in the outcome in this domain in relation to rehabilitation programs followed in Dutch Multidisciplinary Rehabilitation Centers at baseline and follow-up.

**Methods:**

In a cohort of 241 visually impaired persons, the D-AI was assessed at baseline (enrollment), 4 and 12 months, The importance and difficulty of the D-AI goals ‘Handle feelings’, ‘Acceptance’, and ‘Feeling fit’ and difficulty scores of underlying tasks were further analyzed, together with similar or related standardized questionnaires. At baseline, Spearman correlations were determined between D-AI goals and task and additional questionnaires to investigate the construct validity. Corrected and uncorrected linear mixed models were used to determine longitudinal rehabilitation outcomes in relation to rehabilitation programs followed.

**Results:**

Baseline correlations indicated that the difficulty of tasks and the umbrella goal ‘Acceptance’ were not similar. Longitudinal analyses provided insight in some subtle differences in concepts measured at the goal and task level of the D-AI, as well as similar validated questionnaires. After correcting for confounding variables, none of the underlying task difficulty scales changed over time. For goal difficulty scores only ‘Acceptance’ was reported to be significantly less difficult at 4 and 12 months follow-up. Importance scores of goals were stable from baseline to follow-up.

**Conclusion:**

With respect to the constructs measured, results support the formulation of the new goal question ‘Emotional life’ which replaces the goals ‘Handle feelings’ and ‘Acceptance’. Results indicate that MRCs should pay more attention to problems related to mental health. They have started to use the D-AI as it seems a promising tool to investigate and evaluate rehabilitation needs (including those related to mental health) over time and to clearly define rehabilitation goals from the very start.

## Background

The self-reported health-related quality of life of visually impaired persons is lower than that of their sighted peers [1]. Visually impaired persons not only experience limitations in performing (instrumental) activities of daily living [2-6] but also show a loss of activities [7]. Therefore, persons with vision loss experience restrictions in participation in society [8-11] and in maintaining independence and control [12-14].

In addition, persons with low vision show adverse mental health outcomes, such as feelings of social isolation [15], emotional distress [5,16] and depression [17-20]. Similarly, the prevalence of depression in persons with impaired vision is substantial. For example, international studies revealed that 26.9-33.7% of visually impaired older adults has depressive symptoms [20-23] versus 10-15% in the general elderly populations [24]. In addition, visual impairment has been associated with lower (psychosocial) wellbeing, expressed by loss of interest and inability to enjoy activities [25]. Indirectly, the adverse impact on mental wellbeing seems to increase the risk of mortality in persons with a visual impairment [26].

Furthermore, visual impairment has also been associated with fatigue and with a higher probability of concentration problems during entertainment and reading [25]. In addition, having low vision was found to be associated with lower levels of physical activity in leisure time [27] and lower levels of self-reported performance in sports activities compared to a reference population of elderly [8]. Also, persons with a visual acuity of ≤ 0.1 (Snellen) were reported to have significantly more problems with a disturbed sleep/wake rhythm and, subsequently, daytime somnolence which also affects the ability to perform daytime activities [28].

These negative effects of sudden or progressive vision loss also need changes in lifestyle which, in turn, also affect psychological functioning [29]. Although an acquired visual impairment usually causes initial reactions such as shock, denial and depression, there may be a period of adaptation resulting in acceptance of the unwanted situation [30]. In an adjustment process, time seems to be an important factor [31]. However, assessment of adjustment in people with established vision loss indicates that adjustment can be seen as a continuous process, rather than as a process with a definite endpoint [32].This process may be influenced by positive or negative life events (e.g. death of a spouse, birth of a grandchild). Moreover, this process may be positively influenced by feelings of regaining control through having learned new skills (e.g. in rehabilitation programs) or by having received psychological counseling.

Rehabilitation for visually impaired persons often includes a prescription of assistive devices, such as magnifiers, canes or speech-enhanced devices. In the Netherlands, in addition to optometric services, most larger cities have a Multidisciplinary Rehabilitation Center (MRC) for visually impaired persons. MRCs provide additional rehabilitation interventions that focus on, for example, applying environmental changes (e.g. light adaptation at home/work), computer use, activities of daily living (e.g. cooking or self-care) and mobility. Interventions that focus on these latter topics may, indirectly, improve mental (health) aspects that are related to feelings, acceptance and fatigue. In addition, in MRCs, psychologists and social workers also provide group or individual counseling which focus more directly on mental wellbeing. Since there is evidence that mental health aspects (such as depression) may inhibit an optimal rehabilitation outcome [29,33,34] and that better adaptation and adjustment to vision loss is significantly associated with fewer reported functional limitations and with greater improvement after low vision rehabilitation [35], there is growing interest in the psychosocial aspects of vision loss as part of rehabilitation. A meta-analysis of qualitative studies revealed that acceptance of the situation, a positive attitude and social support facilitate the psychosocial adjustment [36], it was advised that the emotional needs of individuals with vision loss should not be neglected and that patients should be referred to counseling and/or peer support groups.

In our previous paper, studying patient files, it was clearly visible that rehabilitation needs related to mental health were often not recognized as being a problem for the patient [37-39]. However, since a study in the Netherlands revealed that 29% of the visually impaired older persons (mean age 78 years, N = 274) entering a MRC showed depressive symptoms, and 7% had a depressive episode over the last year based on the DSM-IV criteria [40], there is a growing awareness in Dutch MRCs that potential psychosocial needs should be investigated, addressed and monitored in visually impaired patients right from the start. In line with this, we developed an instrument to apply a more structured approach to systematically investigate and evaluate rehabilitation from the patient’s perspective which includes topics related to mental health. This new instrument was developed based on Massofs’ ‘Activity Inventory (AI) [41-45].The content of the AI was specifically developed for visually impaired persons across different eye conditions. It has a hierarchical structure in which ‘tasks’ (specific cognitive and motor activities, e.g. ‘place your signature’ or ‘fill in official forms, such as tax forms’) that serve a common purpose are categorized under umbrella (rehabilitation) ‘goals’ (e.g. ‘personal administration’). We translated, extended and adapted the AI [37-39]. Tasks that served a common purpose were put together and named after the goal they served. This process was data driven. Subsequently, all goals were structured according to the Activity and Participation domains of the International Classification of Function, Disability and Health (ICF) [46]. The relevance to detect possible needs related to mental health aspects was recognized by patients, as well as by professionals involved in focus group discussions in the developmental phase of the D-AI [37-39]. Based on their input, an additional domain ‘Coping with mental (emotional) health aspects’ was added to the D-AI which includes the goals ‘Handle feelings’, ‘Acceptance’, and ‘Feeling fit’. It was decided that these goals were not categorized by the domain ‘General tasks and demands’ (i.e. chapter 2 of the ‘Activity and Participation’ domains of the ICF), which includes the topic ‘Handling stress and other psychological demands (other specified/unspecified)’ (d2408/d2409). Items in chapter 2 of the ‘Activity and Participation’ domains of the ICF focused primarily on stress, which did not properly reflect the items of the D-AI that showed up during focus group discussions. In addition, there was consensus (JEB, RMAvN and rehabilitation experts (n = 12)) that the goals ‘Feeling fit’, ‘Handle feelings’ and ‘Acceptance’ had to be mentioned separately, in the last part of the questionnaire, because of the emotional impact these questions may have on the patient. Therefore, a 10th domain (which is not covered by the “Activities and Participation” domains of the ICF) “Coping with mental (emotional) health aspects” was added to the D-AI.

In developing the D-AI, using qualitative techniques in the target population contributed to face and content validity for the D-AI. However, for a better interpretation of the scores, better understanding of the construct validity is also necessary. In this observational study, the D-AI was used to investigate the (longitudinal) interpretation of the concepts in the domain ‘Coping with mental (emotional) health aspects’. Although rehabilitation was not based on the D-AI in this validation study, the data allowed to understand the longitudinal outcomes in this domain in relation to rehabilitation programs followed in Dutch MRCs at 4 and 12 months after enrollment.

## Methods

### Design

A cohort of visually impaired persons was followed and evaluated on the outcome of (vision) rehabilitation at an MRC. The current population was the same as that included in our previous study [47]. All patients were recruited directly after enrollment at the MRC. Measurements were taken at baseline (i.e. before people had the usual intake conversation at the MRC) and at 4 and 12 months follow-up. Data collection took place between May 2008 and March 2010.

### Recruitment of study population

Eligible participants were aged ≥18 years, with adequate command of the Dutch language, and with sufficient cognitive ability to participate in the study (based on observations by the assessor of the D-AI or during the usual intake at the MRC and/or on medical information in the patient file). All patients with a need for low-vision rehabilitation that had enrolled in the MRC were allowed to participate. Persons with low vision from any cause were eligible and there was no restriction regarding visual performance. Although patients usually enter the MRC after referral by an ophthalmologist, patients may be referred by a general practitioner or may enroll in an MRC on their own initiative.

**Figure 1:**
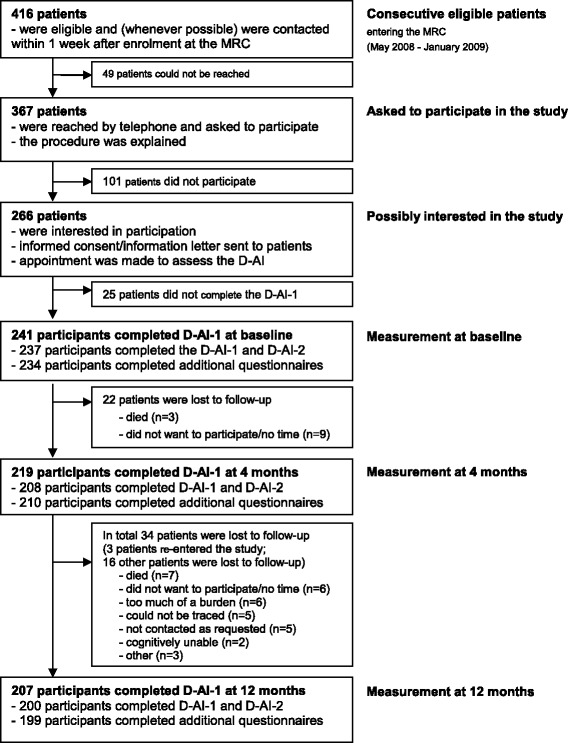
**Flow diagram of study participation: number of patients available at different stages of the study.** D-AI: Dutch ICF Activity Inventory; D-AI-1: goal questions of D-AI; D-AI-2: task questions of D-AI; MRC: Multidisciplinary Rehabilitation Center for visually impaired patients.

As can be seen in Figure 1, consecutive patients who entered the MRC between May 2008 and January 2009 were screened for study eligibility. During that period, of the 416 eligible patients that we tried to reach 367 were contacted, of whom 266 (72.5%) showed an interest in this study [47]. A total of 241 participants completed the D-AI at baseline; of these, 219 and 207 completed the D-AI again at 4 and 12 months, respectively which is described in more detail elsewhere [48]. It was explained to all participants that the D-AI would be assessed in addition to the usual intake procedure at the MRC and that the rehabilitation trajectory would be based on the usual intake at the MRC as their employees were not familiar with the D-AI or its results yet. For the current study, it was only observed when psychosocial care was applied and how importance and difficulty scores changed over time.

The study protocol was approved by the Medical Ethics Committee of the VU University Medical Center Amsterdam and was consistent with the principles of the Declaration of Helsinki. All participants provided written informed consent.

### Outcome measures and data collection

For this ongoing study we used an instrument that was developed based on the Activity Inventory (AI) [41-45], which investigates rehabilitation needs in terms of goals and underlying tasks from the patient’s perspective and enables evaluation of rehabilitation outcome. In a previous study, it was discussed how the AI was updated, extended and adapted to the Dutch situation. It was also described how tasks were collected and grouped into goals and then categorized according to the nine ‘Activity and Participation’ domains of the ICF.[37] The D-AI investigates a broad range of limitations in activities and restriction in participation of visually impaired persons. The full version of the D-AI that was assessed consisted of 65 umbrella goals and 959 underlying tasks. For practical reasons, the D-AI was assessed using a computer-assistive telephone interview [programmed in Blaise Enterprise 4.7 (Heerlen, the Netherlands)] with a routing structure, so that only the most relevant goals were assessed at the task level. First, the first part (‘D-AI-1’) scored the self-reported importance of goals [scale ‘not important’ (0); slightly important (1); moderately important (2); ‘very important’ (3), or ‘not applicable’] and, if the goal was of at least some importance (importance score >0), the self-reported difficulty of goals [scale ‘not difficult’ (0); slightly difficult (1); moderately difficult (2); very difficult (3); ‘impossible’ (4), or ‘not applicable’] was assessed. Subsequently, a priority score was calculated by multiplying goal importance and difficulty. Then, in the second part (‘D-AI-2’), tasks underlying only a limited number of goals that had the highest priority scores (i.e. top 15 priority scores) were assessed using the same difficulty scale [scale ‘not difficult’ (0); slightly difficult (1); moderately difficult (2); very difficult (3); ‘impossible’ (4), or ‘not applicable’] [47]. Therefore, not all items in the current study were assessed equally often, and were not necessarily assessed at each time point for the same participant (e.g. for a participant, a ‘very important’ (3) goal had decreased from ‘impossible’ (4) to ‘not difficult’ (0) from baseline to follow-up; thus the priority score of this goal has decreased from 12 to 0, meaning that underlying tasks were only assessed at baseline).

Previously, we investigated several psychometric properties at baseline and created an improved and shorter version of the D-AI [47]. Exploratory factor analyses (for each goal separately) revealed that the (remaining) underlying tasks underneath the goals in the additional domain ‘Coping with mental (emotional) health aspects’, formed one factor. These scales showed a sufficient to good internal consistency [Cronbach’s alphas: 0.86 (‘Handle feelings), 0.75 (‘Acceptance’), 0.87 (‘Feeling fit’) [47]]. In the present study, the importance and difficulty scores of these goals were used. Moreover, the mean task difficulty score for each scale was calculated for each participant if at least 50% of the tasks in the scale were available (i.e. not ‘not applicable’ or ‘missing’ due to the routing structure). All items can been seen in Table 1.

**Table 1:** **Items used for further analysis**

**DOMAIN 10:**	**‘Coping with mental (emotional) health aspects’**
***Handle feelings***	
***Introduction***	*“The next questions are about your mood and feelings because of your visual impairment”*
**Goal questions**	**How** ***important/difficult*** **is it for you to handle your feelings about your visual impairment?**
**Task questions**	**How** ***difficult*** **is it for you to… [task]… ?**
1	Deal with feelings of loneliness
2	Deal with gloomy or sad feelings
3	Deal with frustration, anger or despair
4	Deal with feelings of anxiety
5	Deal with stress
6	Deal with feelings of inferiority
7	Enjoy joint activities
***Acceptance***	
***Introduction***	*“The next questions are about the handling, acceptance and processing of your visual impairment”*
**Goal questions**	**How** ***important/difficult*** **is it for you to cope with your visual impairment?**
**Task questions**	**How** ***difficult*** **is it for you to… [task]… ?**
1	Be open about your visual impairment with strangers (or in an unfamiliar place)
2	Be open about your visual impairment with acquaintances (or in a familiar place)
3	Deal with incomprehension from others because of your visual impairment
4	Explain others what you can and cannot see
5	Dare to ask for help from persons you know
6	Deny help from persons you know
7	Deal with changing roles and relationships because of your visual impairment (with people close to you)
***Feeling fit***	
***Introduction***	*“The next questions are about problems with fatigue, concentration, and balancing your energy, which may arise from your visual impairment”*
**Goal questions**	**How** ***important/difficult*** **is it for you to feel fit?**
**Task questions**	**How** ***difficult*** **is it for you to… [task]… ?**
1	Sustain your daily activities during the day, such as shopping, cooking or arranging things
2	Finish your daily activities in time
3	Get somewhere without getting too tired
4	Stay focused and concentrated
5	Perform your daily activities without suffering from discomfort in the eyes (e.g. eye strain)
6	Perform your daily activities without suffering from other symptoms (such as neck, back or head)
7	Balance your energy during the day (e.g. so that you have some energy left at the end of the day)
8	Do things in your spare time (such as hobbies or social contacts)
9	Keep a day and night rhythm

“D-AI-1”: Goal questions; Goal Importance scores: not important (0), slightly important (1), moderately important (2), very important (3), not applicable (−); Goal Difficulty scores: not difficult (0), slightly difficult (1), moderately difficult (2), very difficult (3), impossible (4), not applicable (−); Priority score: Goal Importance *Goal Difficulty; “D-AI-2”: Task questions of goals (with a top-fifteen priority score); Task Difficulty scores: not difficult (0), slightly difficult (1), moderately difficult (2), very difficult (3), impossible (4), not applicable (−).

Parallel to the D-AI, several other questionnaires were assessed. First, a short self-report scale was used to measure depressive symptoms over the last week by means of the Center for Epidemiological Studies Depression scale (CES-D) [49]. The sum score ranges from 0 to 60, with higher scores representing more depressive feelings. In the general population respondents with a score of ≥16 are ‘at risk’ for depression [50]. Second, the Dutch version of the Adaptation to Age-related Vision Loss (N-AVL-12) scale was assessed [51-53]. The N-AVL-12 is a measure of psychosocial adjustment specifically developed for older adults who need to adapt to late-life vision loss; it has 12 questions answered on a 4-point scale (0–3), with positive and negative statements (e.g. ‘Because of my vision loss, I feel like I can never really do things for myself’, ‘Because of my trouble seeing, I am afraid that people will take advantage of me’). The sum score ranges from 0 to 36 with higher scores representing a better adaptation to age-related vision loss. With a score ≤14, there is an indication of significant adjustment problems [51-53]. The third questionnaire to be assessed was the Shortened Fatigue Scale (SFQ, in Dutch: *VerkorteVermoeidheidsVragenlijst*, VVV) [54]. The SFQ is a short and simple instrument to determine the intensity of physical fatigue of the patient over the last two weeks. It contains four questions answered on a 7-point scale (i.e. items ‘I feel tired’; ‘I am tired easily’; ‘I feel fit’; ‘I feel physically exhausted’, scaled from ‘yes, that’s right’ to ‘no, that’s not right’). The sum score ranges from 4 to 28 (higher scores represent more physical fatigue) and can be compared to different norm groups. In a sample of Dutch cancer patients receiving radio therapy (mean age 61 year) the middle 33% had scores between 13 and 21, the lower 10% had score 4 and the upper 10% had scores >28. For healthy Dutch adults (mean age 37) these scores were 4, 5–8, and >15,respectively.

Patient characteristics (e.g. age, gender, living condition, educational level, employment, co-morbidity (open ended question “Do you have any other disorders apart from you eye condition?”), use and possession of low vision aids (tick boxes and open space), etc.) were assessed. In addition, the EuroQol 5 dimensions (EQ-5D) was administered to assess the general health status of the participants (on all measurement points) [55-57]. The EQ-5D is a descriptive system of health-related quality of life states, consisting of five dimensions (mobility, self-care, usual activities, pain/discomfort, anxiety/depression). Each of these dimensions has three response options (no problems; some or moderate problems; extreme problems). For every individual a single health state value was calculated where 0 corresponds to death and 1 corresponds to a state of perfect health [56]. Finally, for each participant, supplementary data were collected (retrospectively) from patient files at the MRC; i.e. medical information (visual acuity and eye condition) and additional information about the rehabilitation trajectory (i.e. content and course of rehabilitation, and prescription of low vision aids).

### Data analysis

#### Patient characteristics

Differences between baseline characteristics of patients who were and were not lost to follow-up (at 12 months) were compared using Chi-square tests for categorical variables, independent samples t-tests for continuous variables, and the Mann–Whitney test as a non-parametric alternative.

#### Construct validity

Spearman correlation coefficients were used to measure the strength of the associations at baseline between the D-AI measures and the additional questionnaires. Absolute correlations <0.2 were interpreted as low, correlations of ≥0.2 and <0.4 as medium, and those ≥0.4 as high. We hypothesized low correlations between goal importance questions and standardized questionnaires because these measure different concepts; the opinion of what is of value for an individual is not the same as how difficult or problematic the topic is to achieve or the degree of the symptoms related to the topic. For difficulty scores, we assumed that the correlation between ‘Handle feelings’ and the CES-D, between ‘Acceptance’ and the N-AVL-12, and between ‘Feeling fit’ and the SFQ would be stronger (≥0.1) compared to correlations of these D-AI goals with the other standardized questionnaires. We also hypothesized that the correlation between task difficulty scales and the difficulty of the umbrella goal would be high (≥0.4) and stronger (≥0.1) compared to the difficulty of the other goals in this domain. All variables were expected to be positively correlated.

#### Longitudinal interpretation and outcomes

In this study population, linear mixed models were used to observe longitudinal rehabilitation outcomes for the goals and underlying subscales of ‘Feeling Fit, ‘Handle Feelings, and ‘Acceptance’ of the D-AI, and the CES-D, N-AVL-12, and SFQ scales. The analyses were based on the method of restricted maximum likelihood so that all measurements made at baseline (n = 241), and at 4 months (n = 219) and 12 months (n = 207) could be used in the longitudinal analysis. In this way, data at each time point added to the precision of the estimates. Unstructured patterns or compound symmetry models were applied, depending on the outcome of the difference in the -2log likelihood of the two models. As some variables such as gender [58], visual acuity [4,58,59], education [58], depression [29], and health status [58-60] are known (or assumed) to influence visual functioning and/or longitudinal rehabilitation outcomes, these potential predictors related to personal characteristics were added to the linear models one at a time and, subsequently, the significant variables together, to investigate whether these variables were related to the longitudinal outcomes. Since the EQ-5D was administered at each time point, these measurements were added as time varying covariates. To correct for multiple testing, predictors with a p-value <0.01 were considered to be significant. Longitudinal changes were investigated for corrected and uncorrected outcomes and a p-value of <0.05 was considered significant.

Finally, for corrected models, as an intervention measure, having had ‘(any) training or assistance’ (prescription of low vision aids was not included in this variable) was added as a time varying covariate to better predict the outcomes of rehabilitation. In addition, this variable was split into ‘psychological or psychosocial counseling’, ‘ICT or type training’, ‘communication training’, ‘training for visual devices’, ‘mobility training’, ‘training in activities of daily living or other self-reliance training´ and ‘information/advice/education’ (all time varying covariates). These predictors were added one by one to the corrected longitudinal models. In addition, data on ‘prescription or advice for low vision aids’ were available only for the entire 12-month period (the exact moment was untraceable), and this was added to the model as a predictor. Again, to correct for multiple testing, variables with a p-value <0.01 were considered to be significant. All analyses were performed with SPSS version 20.0 (SPSS IBM, New York, USA).

## Results

### Study population and loss to follow-up

Table 2 presents details on baseline characteristics of patients who did (n = 207) and did not (n = 34) complete the D-AI-1 at 12 months. Participants who were lost to follow-up 12 months after baseline were older, had a lower education level, were less often employed, and reported more health problems (EQ-5D), depressive symptoms (CES-D), problems with adaptation to vision loss (N-AVL-12), fatigue (SFQ) and difficulty to handle feelings (D-AI). Of the respondents (n = 207), 146 (72.6%) patients had comorbidity (self-report), 65 (33.5%) had clinically relevant depressive symptoms (CES-D), and 28 (13.5%) had a suspicion of significant adjustment problems (N-AVL-12). Respondents had a mean fatigue score (SFQ; 13.41 (7.96%).

**Table 2:** **Difference in baseline characteristics between respondents and participants lost to follow-up**

	**Respondents (n = 207)**	**Participants lost to follow-up (n = 34)**	**p-value**
**Gender (female), n (%)**	112 (54.1%)	22 (64.7%)	0.25^§^
**Age in years, median[IQR]**	69.0 [57.0;78.0]	79.0 [73.0;83.3]	<0.001***^‡^
**Education (years), (median [IQR])**	11 [9;15]	9 [6;11]	0.001**^‡^
**Employment**			0.003**^§^
**Employed, n (%)**	42 (21.1%)	0 (0%)	
**Volunteering, n (%)**	30 (15.1%)	2 (6.3%)	
**Not employed or volunteering, n (%)**	127 (63.8%)	30 (93.8%)	
**Residence**			0.11^§^
**Independent living, n (%)**	185 (92.5%)	27 (81.8%)	
**In nursing home, n (%)**	4 (2.0%)	1 (3.0%)	
**Semi-independent, n (%)**	11 (5.5%)	5 (15.2%)	
**Living alone (e.g. no partner, divorced), n (%)**	67 (33.7%)	16 (48.5%)	0.10^§^
**Visual acuity (better eye) in Snellen, mean (SD)**	0.35 (0.29)	0.29 (0.23)	0.26^†^
**Macular degeneration (from patient file), n (%)**	92 (46.5%)	19 (59.4%)	0.18^§^
**Comorbidity (self-reported), n (%)**	146 (72.6%)	27 (81.8%)	0.27^§^
**Use of visual tools (self-report), n (%)**	181 (90.0%)	27 (84.4%)	0.34^§^
**EQ-5D index, mean (SD)**	0.77 (0.20)	0.68 (0.24)	0.01*^‡^
**CES-D, mean (SD)**	12.98 (9.18)	17.81 (12.30)	0.03*^‡^
**CES-D (≥16), n (%)**	65 (33.5%)	18 (56.3%)	0.01*^§^
**N-AVL-12, mean (SD)**	23.11 (7.21)	16.72 (7.72)	<0.001***^†^
**N-AVL-12 (≤14), n (%)**	28 (13.5%)	16 (47.1%)	<0.001***§
**SFQ, mean (SD)**	13.41 (7.96)	19.30 (7.98)	0.003**^‡^
**D-AI**			
**Goal Importance (range: 0–3), mean (SD)**			
**Handle feeling**	2.60 (0.77)	2.65 (0.54)	0.72^‡^
**Acceptance**	2.74 (0.52)	2.67 (0.65)	0.55^‡^
**Feeling fit**	2.89 (0.34)	2.85 (0.44)	0.69^‡^
**Goal Difficulty (range: 0–4), mean (SD)**			
**Handle feeling**	1.32 (1.13)	1.79 (1.20)	0.03*^†^
**Acceptance**	1.59 (1.12)	1.84 (1.22)	0.25^†^
**Feeling fit**	1.32 (1.17)	1.59 (1.21)	0.22^†^
**Task Difficulty (range: 0–4), mean (SD)**			
**Handle feeling**	1.19 (0.82)	1.31 (0.72)	0.62^†^
**Acceptance**	0.96 (0.71)	0.94 (0.69)	0.93^†^
**Feeling fit**	1.13 (0.82)	1.25 (0.60)	0.57^†^

*p <0.05; **p <0.01; ***p <0.001; ^†^: independent t-test; ^‡^: Mann–Whitney U test; ^§^: Chi-square test; Not all data were available for all participants; SD: standard deviation; IQR: Inter Quartile Range; EQ-5D index: EuroQol 5 dimensions index (range [0–1], 0 reflects worst possible health status); CES-D: Center for Epidemiological Studies Depression scale (range [0–60], scores ≥ 16 reflect clinically relevant depressive symptoms); N-AVL-12: Dutch version of the Adaptation to Age-related Vision Loss (12 items, scores ≤14, there is as suspicion of significant adjustment problems); SFQ: Shortened Fatigue Scale (range [4-28], higher scores reflect more fatigue).

### Assistive devices and content of rehabilitation

At baseline, 89.3% of the participants reported to have assistive devices ((mean) number of 2.01 (standard deviation: SD = 1.33)). At 4 and 12 months, 95.2% and 97.0% of the participants reported to have assistive devices ((mean) number of 2.38 (SD = 1.49) and 2.47 (SD = 1.48)), respectively. In the patient files it was reported that within 12 months, to 80.5% of all patients (n = 241) assistive devices were prescribed or advised. Moreover, at 4 months 38 (17.4%) participants and at 12 months 65 (31.4%) participants had received (additional) training (e.g. training for visual devices). Table 3 provides additional variables on the content of rehabilitation at each measurement point. At 12 months, 43 (24.4%) participants reported that more rehabilitation sessions were planned.

**Table 3:** **Type of rehabilitation applied (reported in the patient files)**

**Type of rehabilitation**	**n at M4** ^*^	**% at M4 (n = 219)**	**n at M12** ^*^	**% at M12 (n = 207)**
Any type of assistance/training	38	17.4%	65	31.4%
Psychosocial counseling	2	0.9%	6	2.9%
Psychological counseling	1	0.5%	1	0.5%
Training ‘activities of daily living’	3	1.4%	6	2.9%
Mobility training	9	4.1%	18	8.7%
Orientation and mobility training	0	0.0%	2	1.0%
ICT training	8	3.7%	24	11.6%
Typing and computer training	1	0.5%	8	3.9%
Communication equipment & techniques	0	0.0%	8	3.9%
Training – visual devices	9	4.1%	18	8.7%
Lighting advice	1	0.5%	1	0.5%
Other (self-reliance) training	2	0.9%	9	4.3%
Information	15	6.8%	19	9.2%
Advice/education	15	6.8%	18	8.7%
Employment counseling	0	0.0%	1	0.5%
Leisure support	0	0.0%	3	1.4%

M4: measurement 4 months after baseline; M12: measurement 12 months after baseline.

^*^Data available for all participants (those who were and were not lost to follow-up), however none of the participants who were lost to follow-up in our study had these type of rehabilitation.

### Construct validity

At baseline, the standardized questionnaires showed low correlations with the perceived importance questions of goals. In addition, all other hypotheses concerning the goal ‘Feeling fit’ were accepted. This was not the case for the goals ‘Acceptance’ and ‘Handle feelings’. When comparing the difficulty of goals and underlying tasks with the specific standardized questionnaires, for the goals ‘Acceptance’ and ‘Handle feelings’ the hypothesized correlations were not found. Moreover, for these goals not all hypothesized correlations between task difficulty scales and difficulty of the umbrella goal were confirmed. Table 4 presents more detailed results.

**Table 4:** **Baseline Spearman correlation coefficients for the association between D-AI outcome measures and other measures, and goal difficulty and underlying tasks**

**D-AI items**	**CES-D**	**N-AVL-12**	**SFQ**	**Difficulty score of D-AI goal**	
**Handle feelings**				**Handle feelings scale**	
Goal importance	−0.07	−0.08	−0.14	Goal difficulty Handle feelings:	0.40***
Goal difficulty	0.42***	−0.38***	0.27***	Goal difficulty Acceptance:	0.33**
Handle feelings scale	0.38***	0.26*	0.11	Goal difficulty Feeling fit:	0.20*
**Acceptance**				**Acceptance scale**	
Goal importance	−0.00	0.01	−0.10	Goal difficulty Handle feelings:	0.30***
Goal difficulty	0.37***	−0.42***	0.21**	Goal difficulty Acceptance:	0.22*
Acceptance scale	0.12	−0.05	0.11	Goal difficulty Feeling fit:	0.21*
**Feeling fit**				**Feeling fit scale**	
Goal importance	0.11	0.00	−0.07	Goal difficulty Handle feelings:	0.38***
Goal difficulty	0.43***	−0.24***	0.55***	Goal difficulty Acceptance:	0.33***
Feeling fit scale	0.39***	−0.27**	0.57***	Goal difficulty Feeling fit:	0.54***

*p < 0.05; **p < 0.01; ***p < 0.001; D-AI: Dutch ICF Activity Inventory; CES-D: Centre for Epidemiologic Studies Depression Scale, range [0–60]; N-AVL-12: Dutch version of the Adaptation to Age-related Vision Loss, range [0–36], higher scores represent enhanced adaptation to vision loss; SFS: Short Fatigue Scale [*VerkorteVermoeidheidsVragenlijst*: VVV], range [4-28], higher scores represent more physical fatigue.

### Longitudinal outcomes

For this cohort, Table 5 shows that the importance scores for the goals ‘Handle Feelings’, ‘Acceptance’ and ‘Feeling fit’ remained stable over time. Moreover, there were no significant confounders.

**Table 5:** **Results from longitudinal analyses for domain 10 of the D-AI including change in goal importance, goal difficulty and mean task difficulty, as well as change in CES-D, AVL, and SFQ**

**(D-AI) Outcomes**	**n**	**Mean (SE) BL**	**Mean (SE) 4 M**	**Mean (SE) 12 M**	**95% CI for BL – 4 M**	**95% CI for 4 M – 12 M**	**95% CI for BL – 12 M**	**Predictors (personal characteristics) (p < 0.01)**	**Predictors (Content of Rehabilitation) p < 0.01)**
**D-AI: Handle feelings**									
Goal Importance	241	2.60 (0.05)	2.58 (0.05)	2.64 (0.05)	[−0.10 – 0.14]	[−0.17 – 0.06]	[−0.14 – 0.07]		
(corrected model)	241	2.60 (0.05)	2.58 (0.05)	2.64 (0.05)	[−0.10 – 0.14]	[−0.17 – 0.06]	[−0.14 – 0.07]	-	
Goal Difficulty	240	1.38 (0.08)	1.33 (0.08)	1.29 (0.08)	[−0.11 – 0.20]	[−0.13 – 0.20]	[−0.08 – 0.24]		
(corrected model)	228	1.34 (0.07)	1.32 (0.08)	1.33 (0.08)	[−0.15 – 0.18]	[−0.18 – 0.17]	[−0.16 – 0.18]	EQ5D -; Education -	
Task difficulty (mean)	158	1.21 (0.08)	1.15 (0.08)	1.13 (0.08)	[−0.12 – 0.23]	[−0.16 – 0.20]	[−0.10 – 0.25]		
(corrected model)	154	1.15 (0.07)	1.17 (0.08)	1.15 (0.07)	[−0.20 – 0.16]	[−0.17 – 0.20]	[−0.19 – 0.18]	EQ5D -	
**D-AI : Acceptance**									
Goal Importance	240	2.73 (0.03)	2.75 (0.04)	2.77 (0.04)	[−0.11 – 0.07]	[−0.11 – 0.07]	[−0.13 – 0.05]		
(corrected model)	240	2.73 (0.03)	2.75 (0.04)	2.77 (0.04)	[−0.11 – 0.07]	[−0.11 – 0.07]	[−0.13 – 0.05]	-	
Goal Difficulty	240	1.61 (0.07)	1.33 (0.07)	1.34 (0.08)	[0.13 – 0.44]***	[−0.17 – 0.15]	[0.11 – 0.45]**		
(corrected model)	237	1.58 (0.08)	1.32 (0.07)	1.32 (0.07)	[0.09 – 0.42]**	[−0.16 – 0.18]	[0.09 – 0.44]**	EQ5D -	
Task difficulty (mean)	183	0.93 (0.06)	0.86 (0.06)	0.90 (0.06)	[−0.06 – 0.18]	[−0.17 – 0.09]	[−0.10 – 0.15]		
(corrected model)	179	0.91 (0.06)	0.87 (0.06)	0.92 (0.06)	[−0.10 – 0.16]	[−0.18 – 0.09]	[−0.14 – 0.12]	Age - ; EQ5D -	IAE** ; LVA***
**D-AI : Feeling Fit**									
Goal Importance	241	2.89 (0.02)	2.88 (0.02)	2.87 (0.03)	[−0.05 – 0.06]	[−0.05 – 0.08]	[−0.04 – 0.08]		
(corrected model)	241	2.89 (0.02)	2.88 (0.02)	2.87 (0.03)	[−0.05 – 0.06]	[−0.05 – 0.08]	[−0.04 – 0.08]	-	
Goal Difficulty	241	1.36 (0.08)	1.20 (0.07)	1.23 (0.07)	[0.01 – 0.31]*	[−0.16 - 0.10]	[−0.02 – 0.27]		
(corrected model)	228	1.32 (0.07)	1.24 (0.07)	1.21 (0.07)	[−0.09 – 0.24]	[−0.11 – 0.17]	[−0.05 – 0.26]	Education -; EQ5D -;	
Task difficulty (mean)	170	1.13 (0.07)	1.00 (0.07)	1.08 (0.07)	[−0.01 – 0.28]	[−0.23 – 0.06]	[−0.09 – 0.19]		
(corrected model)	161	1.11 (0.06)	1.03 (0.07)	1.11 (0.07)	[−0.08 – 0.23]	[−0.23 – 0.07]	[−0.16 – 0.15]	Education -; EQ5D -	
**Other questionnaires**									
**CES-D**	239	13.71 (0.62)	12.54 (0.64)	12.71 (0.65)	[0.15 – 2.20]*	[−1.22 – 0.89]	[−0.04 – 2.05]		
(corrected model)	227	13.37 (0.53)	12.73 (0.55)	12.64 (0.56)	[−0.38 – 1.67]	[−0.97 – 1.15]	[−0.31 – 1.77]	Education -; EQ5D -;	
**N-AVL-12**	239	22.23 (0.48)	22.87 (0.49)	22.89 (0.49)	[−1.34 – 0.07]	[−0.73 – 0.69]	[−.1.36 – 0.05]		
(corrected model)	222	22.60 (0.44)	23.20 (0.45)	23.12 (0.46)	[−1.31 – 0.11]	[−0.64 – 0.81]	[−1.24 – 0.20]	Age -; VA +; Education +; EQ5D +	TVD**
**SFQ**	215	14.25 (0.55)	13.00 (0.57)	13.61 (0.56)	[0.37 – 2.13]**	[−1.50 – 0.29]	[−0.21 – 1.49]		
(corrected model)	208	14.04 (0.48)	12.98 (0.51)	13.34 (0.49)	[0.13 – 1.99]*	[−1.31 – 0.59]	[−0.20 – 1.60]	Male -; Education -; EQ5D -;	

*p <0.05; **p <0.01; ***p <0.001; +: positive coefficient; − negative coefficient; BL: Baseline; 4 M: measurement 4 months after baseline; 12 M: measurement 12 months after baseline; SE: standard error; D-AI: Dutch ICF Activity Inventory; CES-D: Centre for Epidemiologic Studies Depression Scale, range [0–60]; N-AVL-12: Dutch version of the Adaptation to Age-related Vision Loss, range [0–36], higher scores represent enhanced adaptation to vision loss; SFS: Short Fatigue Scale [*VerkorteVermoeidheidsVragenlijst*: VVV], range [4-28], higher scores represent more physical fatigue; EQ5D: EuroQol 5 dimensions, range [0–1], 0 means worst possible health status; Education: years of education; VA: Visual Acuity (in Snellen); LVA: prescription of Low Vision Aids, yes = 1, no = 0; IAE: Information/Advice/Education (yes = 1, no = 0); TVD: Training for using Visual Devices, yes = 1, no = 0); LVA: prescription of or advice for assistive device; (variable between brackets): variable was not significant when together with other confounders in one model; First line of each Outcome measure represents the uncorrected model and the second line of each Outcome measure represents the corrected model).

The difficulty of the goal and underlying tasks of ‘Handle feelings’ were stable over time (for corrected as well as uncorrected models). For ‘Acceptance’ the difficulty of the umbrella goal was significantly lower at 4 and 12 months after baseline, for both the corrected and uncorrected models. However, the underlying tasks did not change over time. The difficulty of the goal ‘Feeling fit’ decreased between baseline and 4 months follow-up, but only for the uncorrected model. Four months after baseline, lower difficulty scores of tasks underlying the umbrella goal ‘Feeling fit’ revealed a similar pattern; however, this change was borderline non-significant.

Depressive symptoms expressed by CES-D scores were significantly lower 4 months after baseline, but only for the uncorrected models. Adaptation to vision loss, reflected by the N-AVL-12 scores was stable over time. Fatigue measured by the SFQ decreased between baseline and 4 months follow-up for both the corrected and uncorrected models.

Table 5 shows the longitudinal results in more detail, including significant (p < 0.01) predictors. None of the potential predictors showed a significant interaction with time. With the exception of the D-AI questions on goal importance, all other outcome measures were predicted by general health status (EQ-5D).

Adding ‘(any) additional assistance/training’ to the corrected models showed no interference (p < 0.01) with the outcome measures. However, ‘information/advice’ and ‘prescription of low vision aids’ predicted the outcomes of tasks underlying the goal ‘Acceptance’. In addition, ‘training for using visual devices’ predicted outcomes in the N-AVL-12.

## Discussion

This study evaluated the content of rehabilitation and longitudinal outcomes of ‘Coping with mental (emotional) health aspects’ (i.e. D-AI goals ‘Handle Feelings’, ‘Acceptance’ and ‘Feeling fit’) in a visually impaired population in relation to rehabilitation programs followed in Dutch MRCs, at 4 and 12 months after enrollment. In addition, for the newly developed D-AI used in this observational study, the (longitudinal) interpretation of the concept of ‘Coping with mental (emotional) health aspects’ was investigated using additional questionnaires measuring related or similar constructs (i.e. depression, adjustment to vision loss, and fatigue).

### Observing the content of rehabilitation

Patient files revealed that, during the course of rehabilitation, assistive devices were prescribed or advised to the vast majority (80.5%) of the patients. In addition, 31.4% of the patients received other interventions (Table 3) between the start of rehabilitation and 12 months follow-up. It is remarkable that only a limited number of patients received help directly related to ‘mental health’. However, because for many patients rehabilitation was not yet finished at 12 months follow-up (24.4%), the interventions reported may be incomplete or unfinished.. In addition, as rehabilitation needs were investigated retrospectively, we had to rely on information in the patient’s file; however, these files may not always have been up to date. In addition, it was impossible to link specific interventions to the goal(s) they were supposed to target, as this was not specifically documented. For a better evidence-based practice, transparent documentation of delivered care, a systematic evaluation of rehabilitation needs immediately after enrollment as well as over time, is essential to gain better insight into the effectiveness of rehabilitation [39]. The D-AI seems a promising tool to investigate and evaluate rehabilitation needs over time and to clearly define rehabilitation goals before the start of rehabilitation; however, its use may be further improved when the content of rehabilitation is well documented.

### Interpretation of the constructs being measured

#### ‘Feeling fit’ and physical fatigue

As all hypotheses for the goal ‘Feeling fit’ were accepted, it seems that not only the goal but also its underlying tasks are strongly associated with the additional (physical) fatigue scale. However, both constructs represent a slightly different concept as baseline characteristics for participants and patients who were lost to follow up differed on the SFQ scale (i.e. patients who were lost to follow-up showed much more physical fatigue at baseline compared to respondents), but did not for the D-AI ‘Feeling fit’ scale. Underlying tasks of the ‘Feeling fit scale’ typically represent difficult items for visually impaired persons (as revealed from focus group discussions), providing better insight into vision-related fatigue problems compared to the ‘physical fatigue’ of the SFQ. However, as feedback from assessors revealed that the formulation of the goal question was difficult to understand, we previously suggested to change the formulation of the ‘Feeling fit’ goal question to: “*Some visually impaired persons experience problems with fatigue, concentration, burden, and how to balance energy levels. How important/difficult is this theme for you?*” (see Appendix in [47]). As data collection in the current study had already started, this new formulation should be tested in the future.

#### ‘Handle feelings’ and depression

Correlations for ‘Handle feelings’, indicate that the difficulty of tasks represents the difficulty of the umbrella goal ‘Handle feelings’. Moreover, the construct being measured is moderately (i.e. for tasks) to highly (i.e. for goals) related to depressive symptoms (CES-D). However, the construct of the umbrella goal is similarly related to depressive symptoms (CES-D) and adaptation to vision loss (N-AVL-12). In line with this, data at baseline revealed that patients who were lost to follow-up had more difficulty with ‘Handle feelings’, showed more depressive symptoms (CES-D) and less adaptation to vision loss (N-AVL-12) than respondents. An explanation that ‘Handle feelings’ was related to the CES-D as well as the N-AVL-12 in a similar way, may be that the D-AI does not investigate the feelings themselves, but how the patient is *coping* with these feelings (i.e. ‘How difficult is it for you to handle your feelings about your visual impairment’), which seems to rely on both constructs, i.e. depression and adaptation.

#### ‘Acceptance’ and adaptation to vision loss

Correlations within the goal ‘Acceptance’ indicate that the difficulty of ‘Acceptance’ tasks only moderately reflect the difficulty of the umbrella goal. Moreover, it seems that the construct measured by these underlying tasks is essentially different compared to the construct measured in the additional ‘adaptation’ scale (i.e. N-AVL-12), whereas goal difficulty of ‘Acceptance’ showed strong correlations with this additional scale. This may support the interpretation that these tasks represent a different construct compared to the goal question; the umbrella goal may represent a more intrinsic feeling of acceptance in which time is an important factor. In contrast, not only difficulty of the ‘Acceptance’ goal but also of its underlying tasks did not reveal any difference at baseline between respondents and those who were lost to follow-up, whereas the N-AVL-12 scale indicated that respondents had a better adaptation to vision loss. The N-AVL-12 focuses more on attitudes towards coping strategies and coping style and not so much on the patient’s ability to perform coping strategies with regard to the visual impairment. This may have caused the baseline differences; patients who were lost to follow-up felt less adapted, possibly (partly) due to their lower health status (Table 2).

#### Emotional health aspects

Our previous study [47] revealed that some patients did not understand the difference between the goals ‘Handle feelings’ and ‘Acceptance’ (as formulated in Table 1). Therefore, after consensus based discussions, it was decided to merge both underlying subscales into one umbrella goal ‘Emotional life aspects’, which covers both scales [47]. As the correlation of the two underlying scales (i.e. the items related to ‘Handle feelings’ and to ‘Acceptance’) with the goal question of ‘Handle feelings’ was stronger compared to the correlation with the ‘Acceptance’ goal question, the formulation of the new goal was more similar to the ‘Handle feelings question’. However, the words ‘handle your feelings’ were replaced by ‘handle your emotional life’, to make it applicable to both underlying scales: “*How important/difficult is it for you to handle your emotional life concerning your visual impairment?*” (see Appendix in [47]). Results of the current study support further investigation of this new approach.

### Longitudinal interpretation and outcomes

Opinions on what is of value for a particular patient with regard to ‘Coping with mental (emotional) health aspects’, seemed to be relatively stable over time. Perceived importance scores remained unchanged for all three goals between baseline and follow-up. This is in line with previous findings on the importance scores of goals in the ICF domain ‘Learning and applying knowledge’ (Goals: ‘Reading’, ‘Writing’, and ‘Watching TV’) [47]. The importance of goals probably mainly depends on a patient’s personal factors. However, changes in other factors represented in the ICF framework (i.e. health status or external factors) may also influence the importance of goals for an individual. To evaluate the effectiveness of a specific rehabilitation intervention, a recurrent assessment of importance questions may not be useful. However, to assess needs and compose a rehabilitation plan, the relevance of rehabilitation goals for a particular person should be considered.

#### ‘Handle feelings’ and depression

In general, perceived difficulty to handle feelings (i.e. at both the goal and task level) did not change over time. For uncorrected models, depressive symptoms changed over the first 4 months of rehabilitation; however, this effect disappeared when correcting for educational level and general health status. In addition, treatment did not interfere with perceived difficulty of ‘Handle feelings’, or with depressive symptoms. Burggraaff et al. found similar results on prescription of CCTVs and the effects of training on how to use the device in Dutch MRC [61].

An investigation of low-vision rehabilitation services in the USA revealed that 88% solely offer optical aids with basic training, while psychological services and support groups are offered to 21% and 28%, respectively [62]. In the current study, patient files showed that psychological or psychosocial-related care was applied less frequently; this seems rather low when considering the number of patients with depressive symptoms and high priority scores for the goal ‘Handle feelings’. In a randomized clinical trial, a self-management program resulted in a reduction of depressive symptoms [63-65]. Also, a pilot evaluation of a self-management program for low vision revealed that the program had a positive impact on participants’ mood and their ability to manage the challenges of low vision [66]. Moreover, Rovner et al. reported that patients with age-related macular degeneration benefitted from problem solving therapy [67,68]. In addition, it was found that an integrated mental health and low vision intervention halved the incidence of depressive disorders compared to standard outpatient low vision rehabilitation [69]. These results suggest that counseling can be effective and supports the need to further apply and investigate the effectiveness of interventions that focus on mental health in Dutch MRCs [70] and visual rehabilitation clinics in the UK [71], as well as to study a new care model for integrated depression management in Australia [72].

#### ‘Acceptance’ and adaptation to vision loss

Longitudinal outcomes revealed that perceived difficulty of the umbrella goal ‘Acceptance’ decreased over time. However, the underlying ‘Acceptance’ scale and the adaptation to the vision loss scale (N-AVL-12) were unchanged. As was suggested based on the correlations, the umbrella goal ‘Acceptance’ might represent, another, more general/intrinsic feeling of acceptance in which time is an important factor, i.e. getting used to the idea that being visually impaired will not change, whereas the underlying ‘Acceptance’ scale remains stable because patients do not (yet) incorporate the adaptation in their daily routines. For instance, a patient may still experience the same difficulty to ‘dare to ask for help from persons you know’, but has accepted this as a fact.

Another study using item response models showed that the adjustment measured by the ‘LVQOL-adjustment’ scale had improved in a cohort of visually impaired persons 5 months after baseline but had disappeared after one year [73]. However, this ‘LVQOL-adjustment’ scale represents a slightly different concept, as this scale includes items such as ‘understanding the eye condition’ and ‘visiting friends and family’.

Looking at the content of rehabilitation more specifically, it seems that providing ‘information or advice and education’ resulted in a lower perceived difficulty of tasks underlying the goal ‘Acceptance’. However, it is remarkable that the N-AVL-12 did not detect any change. A closer look at the exact content of the items in the scale ‘Acceptance’ (which encompasses items such as ‘explain to others what you can and cannot see’) may explain the effect of the intervention measured by the ‘Acceptance’ scale; patients receiving ‘information or advice and education’ may be taught how to adapt to the vision loss in specific situations. Since better adaptation and adjustment to vision loss has been significantly associated with greater improvement of vision-related activity limitations after low vision rehabilitation [35], future research should focus on how to further improve adaptation to vision loss.

#### ‘Feeling fit’ and physical fatigue

Longitudinal outcomes of ‘Feeling fit’ revealed that perceived difficulty of the goal decreased between baseline and 4 months follow-up. In addition, in the same period a decrease in perceived difficulty of tasks underlying the goal ‘Feeling fit’ was borderline non-significant. However, after correcting for education and general health status, the umbrella goal, as well as the tasks underneath ‘Feeling fit’, were not significant. In contrast, (physical) fatigue measured by means the SFQ decreased significantly over the same period, also after correcting for confounders (i.e. education, general health status, gender). It seems contradictory that longitudinal changes were different for these outcome measures. A possible explanation is that (as became clear from feedback from assessors in our previous study [47] using the same baseline data as the current study) the goal question of ‘Feeling fit’ was unclear (i.e. it was reported (unpublished data) that ‘How fit do you feel’ would be clearer than ‘How difficult is it for you to feel fit’), however, this would have resulted in different response options compared to the rest of the D-AI. Underlying task questions were reported to be clear. As the content of the D-AI was specifically developed by and for visually impaired persons [37], the underlying ‘Feeling fit’ tasks clearly are vision-related (see Table 1). Another reason why perceived difficulty of ‘Feeling fit’ scale remained unchanged may be that these items partly depend on other concepts, such as ‘physical pain’ (e.g. ‘perform your daily activities without suffering from discomfort in the eyes (e.g. eye strain)’), ‘mobility’ (e.g. ‘go on the road without getting too tired’), and/or ‘mental fatigue’ (e.g. ‘stay focused and concentrated’). In addition, it is possible that patients reported the same perceived difficulty several months after baseline, but in fact performed more daily activities and increased their activity level (also causing weight loss and, in turn, increased fitness) as a result of rehabilitation. It was reported that the reciprocal relationship between activity loss and psychological wellbeing in people with vision impairment means that rehabilitation programs have the potential to be more powerful if both practical skills and psychological factors are addressed concurrently [7]. MRCs may integrate this in their trajectory. In addition, MRCs may offer and/or stimulate visually impaired persons to do more physical training as this increases physical fitness [74] and mental health [75]. As the number of publications related to fatigue in a visually impaired population is limited, use of the D-AI and the results of the current study may make a significant contribution to the literature.

### Study limitations

For most interventions no interference was detected, suggesting that the effect of rehabilitation on mental health aspects was only limited. However, these results should be interpreted with caution as only a few participants were available per treatment. Moreover, baseline characteristics of patients who were and were not lost to follow-up showed a significant difference; therefore, missing data at follow-up should not be considered to be ‘missing at random’ as was assumed in the model. The analyses applied may have caused an underestimation (or overestimation) of the improvement of the difficulty of goals and tasks in this domain. A part of the discrepancy between change patterns in goals and underlying tasks may also be explained by the routing structure, as questions at the task level were rated by only a selection of the participants that rated the goal difficulty question. Moreover, based on the current study design, it is uncertain whether a specific intervention was appropriate and whether it actually caused the improvement. Patients were not randomized into a treatment or control group, but were simply observed during their individual rehabilitation trajectory. Finally, longitudinal measures were interpreted on the assumption that the D-AI was responsive to change, but this assumption might be incorrect. Future research with respect to invariance of measurement is necessary. Although we compared longitudinal measurements in the D-AI with similar constructs, these measures should not be seen as a gold standard. In addition, the number of challenging and specific a priori hypotheses was limited, which increases the risk of bias as it is tempting to reflect on alternative explanations retrospectively [76-78]. The longitudinal validity, as well as the clinically important change, should be investigated more specifically; the current study was not designed for this purpose. Moreover, the mean difficulty scores of tasks in the D-AI domain related to ‘Mental health’ were usually at the bottom of the scale. This may have caused floor effects, resulting in relatively little room for improvement. However, as the current sample not only contains patients with problems related to mental health, these floor effects are probably less profound in a sample of patients especially recruited because of their problems related to mental health.

### Conclusions, clinical implications and future research

Based on the current results and of our previous study [47], some adaptations were made to the domain ‘Coping with mental (emotional) health aspects’ of the D-AI. These changes need to be investigated more thoroughly. When more data become available, we will apply additional analyses (e.g. using item response models) to further improve the validity, reliability and interpretability of the newly formulated questions of the D-AI.

Baseline scores for the additional domain ‘Coping with mental (emotional) health aspects’ of the D-AI underscore that visually impaired persons experience mental health problems related to their vision loss and that they might need and want help for this. In addition, although it is possible that the effect of rehabilitation was not yet fully achieved as rehabilitation was not yet finished for many patients, longitudinal outcomes showed only limited improvement on ‘Acceptance’, which should at least partly be attributed to a natural adaptation to vision loss. Other goals and tasks remained unchanged. These results indicate that MRCs should pay more attention to problems related to mental health. The D-AI may help to recognize needs related to mental health from the very start by asking patients more directly about their vision-related mental health. Moreover, the detailed tasks of the D-AI may facilitate the monitoring of mental health status over time in relation to vision loss.This was an important reason for Dutch MRCs to start using the D-AI as part of the standard assessment of rehabilitation needs intake and evaluation procedure. In addition, evaluation of the effectiveness of new rehabilitation interventions related to mental health aspects is also warranted. The D-AI can be useful to study newly developed rehabilitation strategies to refer patients to the right rehabilitation trajectory and to monitor outcome with respect to vision-related mental health outcomes. However, the D-AI mental health scales serve a different purpose compared to psychological diagnostic scales that are already available.
